# Executive Function Strengths in Athletes: a Systematic Review and Meta‐Analysis

**DOI:** 10.1002/brb3.70212

**Published:** 2024-12-31

**Authors:** Shuangquan Ren, Peng Shi, Xioasu Feng, Kai Zhang, Wenchao Wang

**Affiliations:** ^1^ Department of Physical Education Northeast Petroleum University Daqing China; ^2^ School of Physical Education Shanghai University of Sport Shanghai China; ^3^ School of Physical Education Liaoning Normal University Dalian China; ^4^ School of Athletic Performance Shanghai University of Sport Shanghai China; ^5^ School of Physical Education Shandong University of Aeronautics Binzhou China; ^6^ School of Graduate Studies Shenyang Sport University Shenyang China

**Keywords:** athletes, brain sciences, cross‐sectional study, executive function, sport skills, sports experience

## Abstract

**Objective:**

Whether athletes possess superior executive functions still needs further examination. Therefore, the aim of this study is to explore the executive function advantages of athletes and the differences in these advantages between open‐ and closed‐skill sports through systematic review and meta‐analysis.

**Methods:**

Computer searches of CNKI, Web of Science, PubMed, ScienceDirect, and SPORTDiscus databases were conducted. After document selection, data extraction, and quality assessment by two researchers, data processing, statistical analysis, and visual presentation were performed using SPSS 25.0, Stata 16.0, and GraphPad Prism 8 software.

**Results:**

A total of 41 articles were included, including 3845 athletes with a mean age of 9.6–42.8 years. Athletes showed more positive inhibitory control (*Z *= 5.18, standardized mean difference (SMD) = −0.631, 95% confidence intervals (*CI*) = −0.869 to −0.392, *p *= 0.000) and working memory (*Z *= 3.42, *SMD *= −0.382, 95%*CI *= −0.601 to −0.163, *p *= 0.001) compared to the general group with no sports experience. Elite and sub‐elite, and sub‐elite and amateur athletes all showed more positive performance on the cognitive flexibility task compared to the latter. In addition, open‐skilled athletes performed more positively on working memory and cognitive flexibility tasks compared to closed‐skilled athletes. Egger linear regression analysis revealed a possible publication bias for inhibitory control, whereas there was no publication bias for working memory and cognitive flexibility. Univariate meta‐regression analysis revealed that date of publication (*β *= 0.145) and sample size (*β *= −0.002) were sources of heterogeneity between studies for the inclusion of cognitive flexibility (*p *< 0.05). The sensitivity analysis of the one‐by‐one elimination method and the cut‐and‐patch method found the results to be relatively robust and reliable.

**Conclusion:**

Athletes have superior executive function performance that increases with sports experience. In addition, open‐skilled athletes showed more positive executive function. The result has guiding significance for the selection and training of athletes in the future.

## Graphical abstract

Athletes showed more positive inhibitory control and working memory compared to the general group with no sports experience. Elite and sub‐elite, and sub‐elite and amateur athletes all showed more positive performance on the cognitive flexibility task compared to the former. In addition, open‐skilled athletes performed more positively on executive function, whereas closed‐skill athletes performed more positively on the inhibitory control task only.

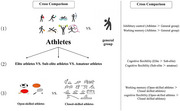

## 1 Introduction

Executive functions are a set of top‐down, higher order cognitive processes that are closely associated with the activity of the prefrontal cortex (Ferguson et al. 2021; Friedman and Robbins 2022). They are primarily used to regulate and manage other cognitive processes in order to achieve goal‐directed behavior (Perone et al. 2021). Executive functions are widely recognized as a multidimensional structure (Baddeley 1996), consisting mainly of three components: inhibitory control, working memory, and cognitive flexibility (Miyake et al. 2000; Pennington and Ozonoff 1996). Although executive functions also encompass higher order functions such as planning and problem‐solving, most research in practice focuses on the aforementioned three basic components (Best and Miller 2010). Executive functions play an important role in real‐life decisions, reasoning, and building of good interpersonal relationships (Devine et al. 2016; Zelazo et al. 1997). Therefore, the study of facilitation strategies for executive functions is important for the future development of individuals.

Executive functions also play a crucial role in regulating athletes’ sports decision‐making and performance. First, in sports contexts, athletes need to consciously filter key information, inhibit irrelevant information, and store this information in the working memory system, matching it with the long‐term memory system to flexibly select the corresponding motor programs for effective decision‐making (Shi et al. 2023). Executive functions are important in visual search and sports decision‐making to make the most appropriate response to stimuli (Schiebener et al. 2013). Research (Li and Chi 2017) has confirmed that the better the athletes’ executive functions, the more prominent their information processing abilities, which is more conducive to improving decision‐making quality. Moreover, if executive functions are impaired, it often leads to an inability to cognitively process targets, severely affecting the decision‐making process and resulting in a decline in decision‐making quality (Hughes 2002). Second, studies (Pageaux and Lepers 2018; Smith et al. 2018) have found that the reduction in agility, coordination, and other sports performances in competitive situations is related to executive functions impairment caused by mental fatigue. Therefore, developing athletes’ executive functions has positive significance for enhancing their sports decision‐making and performance.

Sports experience refers to the multidimensional integrated experience of knowledge, skills, cognition, and emotions that individuals accumulate through interaction with the external world using their bodies (Liu and Jiao 2022). Reflected at the cognitive level of sport skills, this experience mainly includes declarative and procedural knowledge obtained in specific sports, as well as the effectiveness of visual search during sports, the rationality and creativity of decision‐making, and so on (García‐Ceberino et al. 2020; Shi et al. 2023). The richer an athlete's sports experience, the more opportunities they typically have to participate in competitions at different levels, thereby having the opportunity to achieve a higher level of sports proficiency. At the same time, a higher level of sports proficiency also reflects the athlete's high level in terms of specific skills, tactical understanding, and psychological quality, all of which are obtained through long‐term sports practice and experience accumulation. Therefore, researchers (Jin et al. 2023; Shi et al. 2023) often use the level of sports proficiency to measure the level of sports experience. On the basis of the level of sports experience, researchers (Fleddermann et al. 2023; Huijgen et al. 2015) usually classify athletes into three categories: elite, sub‐elite, and amateur. Elite athletes typically refer to those who can compete at a high level of competition. They usually have the highest competitive level and can represent their country or region in competitions. Sub‐elite athletes have a competitive level slightly lower than elite athletes. They may perform well in national or regional level competitions but have not yet reached the top level. Amateur athletes usually refer to those who participate in sports activities in their spare time and have relatively lower sports experience.

Athletes continuously adapt to the external environment during training and competition, accumulating a wealth of sports experience, and demonstrating autonomous physical and psychological skills, such as rapid information processing, effective attention allocation, and efficient sports decision‐making (Fleddermann et al. 2023). Researchers have primarily used cross‐sectional studies to explore the cognitive task advantages of athletes. For example, related studies (Chang et al. 2017; Fleddermann et al. 2023; Heppe et al. 2016; Logan et al. 2023; Scharfen and Memmert 2019) have shown that elite athletes outperform the general population in general cognitive tasks such as sustained attention, attention allocation, response inhibition, and cognitive flexibility. However, related studies are controversial, for example, Logan et al. (2023) showed that there was no significant difference in inhibitory control between athletes and controls; Heppe et al. (2016) showed that there was no significant difference in processing speed and memory breadth between elite athletes compared to amateurs; and Holfelder et al. (2020) showed that the hot executive functions of elite and amateur athletes do not differ significantly. Even a recent systematic review and meta‐analysis (Kalén et al. 2021) showed that elite athletes performed lower on cognitive tasks than amateurs.

The relevant systematic reviews and meta‐analyses aim to resolve this controversy by comprehensively sorting out the literature in the field and combining effect tests. Systematic reviews and meta‐analyses (Albaladejo‐Garcia et al. 2023; Heilmann et al. 2022; Kalén et al. 2021; Liu et al. 2024; Logan et al. 2023; Sabarit et al. 2022; Schaufen and Memmert 2019; Voss et al. 2010) on similar topics have confirmed that athletes possess higher cognitive skills, particularly in attention allocation (Logan et al. 2023), inhibitory control (Albaladejo‐Garcia et al. 2023), cognitive flexibility (Logan et al. 2023), and multi‐object tracking (Liu et al. 2024). However, there are still some limitations in the aforementioned studies. First, although some studies (Albaladejo‐Garcia et al. 2023; Logan et al. 2023) have explored the inhibitory control and cognitive flexibility of executive functions, there is insufficient exploration of working memory, thus preventing a comprehensive understanding of the role of executive functions in sports experience. Second, researchers (Albaladejo‐Garcia et al. 2023; Heilmann et al. 2022; Liu et al. 2024; Logan et al. 2023; Sabarit et al. 2022; Schaufen and Memmert 2019) mostly compare athletes with a general control group, and the included athlete groups encompass elite, sub‐elite, and amateur athletes, neglecting the heterogeneity of athletes with different experiences. In addition, although one study (Logan et al. 2023) has categorized athletes, they only compare different experience groups with a general control group, neglecting the horizontal comparison among elite, sub‐elite, and amateur athletes. Therefore, the relationship between sports experience and executive functions task performance has not been sufficiently demonstrated.

Finally, the differences between open‐ and closed‐skill athletes and a general control group need further examination. There are skill‐type differences in the benefits that exercise produces on cognition, which may be related to the motor task of the activity it involves (Chang et al. 2017). An enriched environment promotes the secretion of brain‐derived neurofactors, establishes effective synaptic connections, facilitates synaptic remodeling, and promotes brain development (Mansour et al. 2021; Nithianantharajah and Hannan 2006). As a result, sport skills are also mostly categorized based on the predictability of the environmental context in studies exploring the relationship between sport skill type and executive functions (Becker et al. 2018; Chueh et al. 2017; Ludyga et al. 2022; Yu et al. 2017; Zhu et al. 2020). On the basis of the unpredictability of the environmental context, sport skills can be categorized into open and closed skills, where the former refers to skills that perform movement tasks in unpredictable environments that require individuals to react and adjust their movements according to changes in the environment, and the latter refers to skills that perform movement tasks in stable, predictable environments where individuals are able to plan their movement routines in advance (Zhang 2012). Within this conceptual framework, a large body of research (Ludyga et al. 2022; Yu et al. 2017; Zhu et al. 2020) supports the idea of the enriched environment hypothesis, which states that open skills intervene better than closed skills in executive functions. However, Heilmann et al. (2022) do not support the aforementioned viewpoint. Their study found no significant differences in inhibitory control, working memory, and cognitive flexibility between open‐ and closed‐skill athletes. Yet, on the basis of theoretical extrapolation, this issue still requires further examination.

On the basis of the limitations of the above studies, this study systematically searched for original studies on cross‐sectional comparisons of executive functions between athletes and the general group and used meta‐analysis for quantitative testing. On the basis of the previous research, this study proposes the following hypotheses:

H1: The executive functions of athletes are superior to those of the general population with no sports experience;
H2: With the increase in the level of sports proficiency, the executive functions of athletes also improve;
H3: Athletes trained in open skills demonstrate more positive performances in executive function tasks.

Through this study, first, it is expected to confirm the role of sports experience in executive functions and provide cognitive evidence for professional skill learning in children and adolescents; second, it is expected to confirm the heterogeneity of skill types and provide theoretical support for further improving exercise intervention programs for executive functions; finally, it contributes to the screening role of executive functions, providing a psychological dimension for the selection and training of athletes.

## 2 Methods

This study was registered (CRD42023460758) in the International Prospective Register of Systematic Reviews (PROSPERO). The Preferred Reporting Items for Systematic Reviews and Meta‐Analyses (PRISMA 2020) guidelines were followed for this study (Page et al. 2021).

### 2.1 Search Strategies

One researcher conducted a relevant literature search using Chinese and English search terms. The study was searched using the following two sets of search terms: (1) “executive function,” “working memory,” “inhibition control,” “inhibitory control,” “cognitive flexibility,” “self‐control,” “self‐regulation”; (2) “athletes,” “player,” “sports grade,” “sports level,” “sports experience,” “years of exercise,” “sports frequency,” “exercise frequency.” This study used the Boolean logic operator AND to concatenate two sets of search terms in the CNKI, Web of Science (WOS), PubMed, ScienceDirect, and SPORTDiscus databases, with a timeframe of the establishment of this database until November 2022. In addition, this study also utilized Google Scholar to conduct further searches for related articles; moreover, it reviewed the references of included studies to avoid any omissions.

### 2.2 Selection Criteria

The study was designed with the following inclusion and exclusion criteria according to the PICOS principles (Costantino et al. 2015). Inclusion criteria: (1) able‐bodied athletes of all levels who specialize in the training and competition of particular sport; (2) athletes participate in training and competition in specific specialized sport skills; (3) the control group was a general population with no or little participation in training and competition in specific sport skills; (4) outcome variables included inhibitory control, working memory, and cognitive flexibility; and (5) the study design was a cross‐sectional study (CSS). Exclusion criteria: (1) athletes with disabilities; (2) studies that did not report or could not identify the type of sport skill, for example, the original study included athletes with different skill types, including ball games and track and field; (3) longitudinal studies; (4) reviews, abstracts, letters, comments, and so on; (5) studies lacking raw data (mean and standard deviation); and (6) duplicate publications for the same study population, and only articles of relatively high quality were included. Screening was carried out independently by two researchers, and the screened literature was secondarily assessed by two other researchers, and if there was a controversy, the group discussion was mutually agreed upon.

### 2.3 Data Extraction

Extracted data included bibliographic information (first author and publication date), participants (sample size, age, and proportion of females), exposure and control measures, and outcome variables (test instruments and main findings). In addition, this study categorized the specialized skills of athletes based on the classification system of sport skills (Zhang 2012). This study entered the extracted information into Excel 2010 and saved it. The included studies used three types of evaluation indexes: reaction time, accuracy, and score, to reflect the execution function of the participants; the faster the reaction time, the higher the accuracy, and the higher the score, the better the execution function of the participants. To ensure the consistency of the direction of the assessment indexes, the study coded the accuracy rate and score value by inverse extraction. Data extraction was carried out by two researchers independently of each other, and the extraction was assessed twice by the other two researchers, and if there were controversial issues, a group discussion was held to decide together.

### 2.4 Quality Assessment

The Agency for Healthcare Quality and Research (AHRQ) scale (Bindman 2017) was used to assess the quality of CSS. The tool, developed by the US AHRQ to assess the quality of cross‐sectional studies, consists of 11 entries, with the evaluator asked to rate each entry using “yes,” “no,” and “unclear.” Given that the included studies were all human trials and there was no follow‐up, entries 4 and 11 were excluded, and 9 entries were used for the quality assessment. Judgments were made by two researchers independently of each other based on the assessment tool, and if there were serious disagreements on the entries, they were discussed with a third researcher.

### 2.5 Statistical Methods

This study used Stata 16.0 and GraphPad Prism 8 software for data processing and statistical analysis. Meta‐analysis used standardized mean difference (SMD) to represent effect sizes and 95% confidence intervals (CI) to represent intervals of estimation of the overall parameters constructed from the sample statistics. This study used the *Q* test and *I^2^* statistic to test inter‐study heterogeneity. If *I* ^2^ < 50% and *p *> 0.1, inter‐study heterogeneity was considered small and a fixed‐effects model was chosen for the analysis; if *I* ^2^ ≥ 50% and *p *≤ 0.1, inter‐study heterogeneity was considered large and a random‐effects model was chosen for the analysis (Shi and Li et al. 2022). This study explored the differences in executive functions of athletes by skill types through subgroup analyses. In addition, this study used Egger's linear regression model for publication bias test; one‐way meta‐regression analysis to explore the sources of heterogeneity; and sensitivity analyses using the one‐by‐one exclusion method and the cut‐and‐patch method. This study set the heterogeneity test level at *α *= 0.1 and the rest of the test level at *α *= 0.05.

## 3 Results

### 3.1 Literature Search Results

**FIGURE 1:**
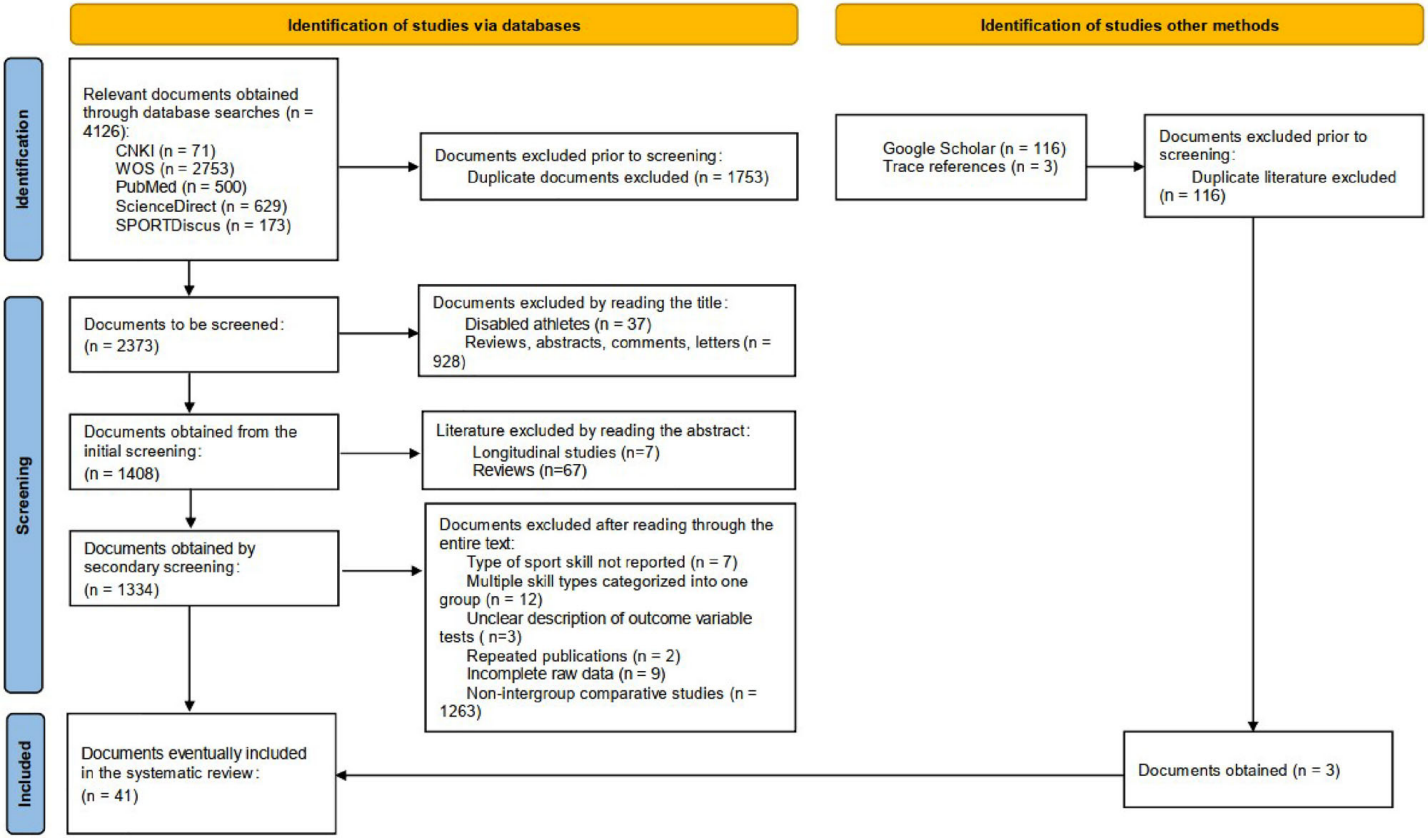
Flowchart used for literature screening. WOS, Web of Science.

A total of 4126 articles were retrieved in this study, and the retrieved articles were imported into EndNote X9 software for de‐duplication, resulting in 2373 articles. After screening, a total of 41 articles were included. The screening process is shown in Figure 1.

### 3.2 Basic Information on Included Studies

#### 3.2.1 Bibliographic Information

Out of 41 articles, the publication years range from 2008 (Nakamoto and Mori 2008a, 2008b) to 2022 (Wu, Xu et al. 2022; Yongtawee et al. 2022). Among them, there are 13 articles published from 2008 to 2015 (Alves et al. 2013; Chan et al. 2011; Cona et al. 2015; Furley and Memmert 2010; Huijgen et al. 2015; Nakamoto and Mori 2008a, 2008b; Seo et al. 2012; Verburgh et al. 2014; Vestberg et al. 2012; Wang, Chang, Liang et al. 2013; Wang, Chang, Liang, Shih et al. 2013; Wang et al. 2015), accounting for 31.71%. There are 28 articles published from 2016 to 2022 (Alarcón et al. 2017; Bashore et al. 2018; Beavan et al. 2019; Bianco and Berchicci et al. 2017; Bianco and Di Russo et al. 2017; Chang et al. 2017; De Waelle et al. 2021; Elfering‐Gemser et al. 2018; Gökçe et al. 2021; Kruger et al. 2019; Liao et al. 2017; Lundgren et al. 2016; Meng et al. 2019; Rodrigues et al. 2019; Sakamoto et al. 2018; Shao et al. 2020; Stacey et al. 2021; Terpstra et al. 2019; van de Water et al. 2017; Vaughan and Laborde 2021; Vestberg et al. 2020; Yongtawee et al. 2022; Wang et al. 2016; Wu, Xu et al. 2022; Wylie et al. 2018; Yu et al. 2017, 2019; Yu and Liu 2021), accounting for 68.29%.

#### 3.2.2 Participants Characteristics

A total of 41 articles included 3845 participants for analysis. The minimum number of participants was 18 (Nakamoto and Mori 2008a), and the maximum number was 383 (Sakamoto et al. 2018). The number of athletes was 2991, accounting for 77.79%, and the number of controls was 854, accounting for 22.21%. Except for two studies (Nakamoto and Mori 2008a, 2008b), the age of the participants was mentioned. The average age of the participants ranged from 9.8 years old (Sakamoto et al. 2018) to 55.0 years old (Terpstra et al. 2019). Seven articles (Alves et al. 2013; Beavan et al. 2019; De Waelle et al. 2021; Huijgen et al. 2015; Sakamoto et al. 2018; Verburgh et al. 2014; Wu et al. 2022) focused on children and adolescent athletes, accounting for 17.95%; the remaining 32 articles (82.05%) focused on adult athletes. Except for Nakamoto and Mori (2008a) and Lundgren et al. (2016), the remaining 39 studies mentioned the gender ratio of the participants. A total of 18 articles (Alarcón et al. 2017; Bashore et al. 2018; Beavan et al. 2019; Cona et al. 2015; Furley and Memmert 2010; Huijgen et al. 2015; Kruger et al. 2019; Nakamoto and Mori 2008b; Rodrigues et al. 2019; Sakamoto et al. 2018; Stacey et al. 2021; Terpstra et al. 2019; van de Water et al. 2017; Verburgh et al. 2014; Wang, Chang, Liang et al. 2013; Wang, Chang, Liang, Shih et al. 2013; Wylie et al. 2018; Yongtawee et al. 2022) focused solely on male athletes, accounting for 46.15%; 3 articles (De Waelle et al. 2021; Seo et al. 2012; Wang et al. 2015) focused solely on female athletes, accounting for 7.69%; and 18 articles (Alves et al. 2013; Bianco and Berchicci et al. 2017; Bianco and Di Russo et al. 2017; Chan et al. 2011; Chang et al. 2017; Elfering‐Gemser et al. 2018; Gökçe et al. 2021; Liao et al. 2017; Meng et al. 2019; Shao et al. 2020; Vaughan and Laborde 2021; Vestberg et al. 2012, 2020; Wang et al. 2016; Wu et al. 2022; Yu et al. 2017, 2019; Yu and Liu 2021) did not distinguish the gender of the athletes, accounting for 46.15%. Among them, the proportion of female athletes ranged from 8.3% (Bianco and Berchicci et al. 2017) to 60.0% (Elferink‐ Gemser et al. 2018).

#### 3.2.3 Exposure and Control Measures

The included studies comprise 12 studies focusing on soccer players (Bashore et al. 2018; Beavan et al. 2019; Huijgen et al. 2015; Rodrigues et al. 2019; Sakamoto et al. 2018; Terpstra et al. 2019; Verburgh et al. 2014; Vestberg et al. 2012, 2020; Wu et al. 2022; Wylie et al. 2018; Yongtawee et al. 2022), 6 studies on badminton players (Liao et al. 2017; Meng et al. 2019; van de Water et al. 2017; Wang et al. 2015; Yu et al. 2017, 2019), and 4 studies on basketball players (Alarcón et al. 2017; Furley and Memmert 2010; Nakamoto and Mori 2008b; Vaughan and Laborde 2021). In addition, there are three studies each on fencers (Bianco and Di Russo et al. 2017; Chan et al. 2011; Gökçe et al. 2021) and swimmers (Gökçe et al. 2021; Wang, Chang, Liang et al. 2013; Wang, Chang, Liang, Shih et al. 2013); two studies each on track and field athletes (Yu et al. 2017, 2019), baseball players (Nakamoto and Mori 2008a, 2008b), tennis players (Wang, Chang, Liang et al. 2013; Wang, Chang, Liang, Shih et al. 2013), volleyball players (Alves et al. 2013; Meng et al. 2019), table tennis players (Elferink‐Gemser et al. 2018; Wang et al. 2016), boxers (Bianco and Di Russo et al. 2017; Yongtawee et al. 2022), marathon runners (Chang et al. 2017; Cona et al. 2015), and shooting athletes (Shao et al. 2020; Yongtawee et al. 2022); and one study each on ice hockey players (Lundgren et al. 2016), martial arts set athletes, rugby players (Kruger et al. 2019), Brazilian jiu‐jitsu practitioners (Stacey et al. 2021), and archers (Seo et al. 2012). There are also three studies that did not focus on athletes of a single skill but included athletes with multiple skills (Bianco and Berchicci et al. 2017; De Waelle et al. 2021; Yu and Liu 2021).

This study, based on the perspective of sport skill classification (Zhang 2012), divides athletes into open‐ and closed‐skill athletes. Among them, 42 studies (Alarcón et al. 2017; Alves et al. 2013; Bashore et al. 2018; Beavan et al. 2019; Bianco and Berchicci et al. 2017; Bianco and Di Russo et al. 2017; Chan et al. 2011; De Waelle et al. 2021; Elfering‐Gemser et al. 2018; Furley and Memmert 2010; Huijgen et al. 2015; Gökçe et al. 2021; Kruger et al. 2019; Liao et al. 2017; Lundgren et al. 2016; Meng et al. 2019; Nakamoto and Mori 2008a, 2008b; Rodrigues et al. 2019; Sakamoto et al. 2018; Stacey et al. 2021; Terpstra et al. 2019; Vaughan and Laborde 2021; van de Water et al. 2017; Verburgh et al. 2014; Vestberg et al. 2012, 2020; Wang, Chang, Liang et al. 2013; Wang, Chang, Liang, Shih et al. 2013; Wang et al. 2016, 2015; Wu et al. 2022; Wylie et al. 2018; Yongtawee et al. 2022; Yu et al. 2017, 2019; Yu and Liu 2021) have explored open‐skill athletes; 11 studies (Chang et al. 2017; Cona et al. 2015; De Waelle et al. 2021; Gökçe et al. 2021; Seo et al. 2012; Shao et al. 2020; Wang, Chang, Liang et al. 2013; Wang, Chang, Liang, Shih et al. 2013; Yongtawee et al. 2022; Yu et al. 2017, 2019) have explored closed‐skill athletes.

A total of 15 articles have cross‐sectional comparisons of executive function in elite, sub‐elite, and amateur athletes (Alarcón et al. 2017; Beavan et al. 2019; Cona et al. 2015; Elferink‐Gemser et al. 2018; Huijgen et al. 2015; Kruger et al. 2019; Lundgren et al. 2016; Sakamoto et al. 2018; Shao et al. 2020; van de Water et al. 2017; Vaughan and Laborde 2021; Verburgh et al. 2014; Vestberg et al. 2012, 2020; Wu et al. 2022); and 29 articles cross‐sectional comparisons of executive function in athletes and general controls (Alves et al. 2013; Bianco and Berchicci et al. 2017; Bianco and Di Russo et al. 2017; Bashore et al. 2018; Chan et al. 2011; Chang et al. 2017; De Waelle et al. 2021; Furley and Memmert 2010; Gökçe et al. 2021; Liao et al. 2017; Meng et al. 2019; Nakamoto and Mori 2008a, 2008b; Rodrigues et al. 2019; Shao et al. 2020; Seo et al. 2012; Stacey et al. 2021; Terpstra et al. 2019; Vaughan and Laborde 2021; Verburgh et al. 2014; Wang, Chang, Liang et al. 2013; Wang, Chang, Liang, Shih et al. 2013; Wang et al. 2016, 2015; Wylie et al. 2018; Yongtawee et al. 2022; Yu et al. 2017, 2019; Yu and Liu 2021). The control measure included in the study was the absence of any specific skill training. Among them, 29 studies established a control group with no specific training experience, and the detailed results can be seen in Table 1.

**TABLE 1:** Basic information on included studies.

Included studies	Participant characteristics (sample size/age/*F*%)	Exposure and control measures	Outcome variables	
			Tools	Results
Wu et al. (2022)	*E*1 = 48/14.81*y*/100.0% *E*2 = 42/16.94*y*/11.9%	Soccer (OP) municipal team players, average training 4.55 years, 6 times/week, 180 min/session (*E*1) vs. soccer (OP) specialized students, average training 3.84 years, 5 times/week, 90 min/session (*E*2)	① Stroop (*E*1 = *E*2) ② 1‐back (*E*1 > *E*2) ③ More‐odd shifting (*E*1 > *E*2)	& & &
Yu et al. (2019)	*E*1 = 18/21.1 ± 2.2*y*/55.6% *E*2 = 18/21.1 ± 2.0*y*/61.1% *N *= 18/21.8 ± 2.1*y*/50.0%	Badminton (OP) college athletes, 11.3 ± 2.7 years of training (*E*1) vs. track and field (CL) college athletes, 7.9 ± 1.6 years of training (*E*2) vs. general college students without specialized training (*N*)	① TSWT (*E*1 > *E*2) ③ CTT (*E*1 = *E*2)	+& +&
Huijgen et al. (2015)	*E*1 = 47/15.5 ± 0.9*y*/0.0% *E*2 = 41/15.2 ± 1.2*y*/0.0%	Soccer (OP) elite athletes, belonging to the top 0.5% of the National Soccer Association (*E*1) vs. soccer (OP) sub‐elite athletes, belonging to the top 12.5% of the National Soccer Association (*E*2)	① SST (*E*1 >* E*2) ② Backword VMS (*E*1 = *E*2) ③ TMT‐B (*E*1 >* E*2)	& & &
Chan et al. (2011)	*E *= 30/20.6 ± 2.1*y*/50.0% *N *= 30/20.6 ± 2.1*y*/50.0%	Fencing (OP) athlete with more than 5 years of training experience, 5–6 times/week (*E*) vs. inexperienced college students (*N*)	① GO/NO GO	0
Nakamoto and Mori (2008a)	*E *= 9/NC/NC *N *= 9/NC/NC	Baseball (OP) college athletes (*E*) vs. college students with no sports experience (*N*)	① GO/NO GO	+
Vestberg et al. (2012)	*E*1 = 29/25.3 ± 4.2*y*/51.7% *E*2 = 28/22.8 ± 4.1*y*/39.3%	Soccer (OP) elite athletes from the highest Swedish soccer league (*E*1) vs. soccer (OP) sub‐elite athletes from the 2nd and 3rd Swedish leagues (*E*2)	① Stroop (*E*1 >* E*2) ③ TMT‐B (*E*1 >* E*2) ③ Design fluency (*E*1 >* E*2)	& & &
Wang, Chang, Liang et al. (2013)	*E*1 = 20/20.4 ± 2.7*y*/0.0% *E*2 = 20/19.3 ± 0.8*y*/0.0% *N *= 20/20.4 ± 2.1*y*/0.0%	Tennis (OP) school team members, 3 to 11 years of training experience, more than 3 times/week, 3 h/session (*E*1) vs. swimming (CL) school team members, 2.5–9 years of training experience, 5 times/week, 2.5h/session (*E*2) vs. students with no sport specialty (*N*)	① SST (*E*1 >* E*2 = N)	+0&
Furley and Memmert (2010)	*E *= 54/24.8 ± 2.7*y*/0.0% *N *= 58/24.8 ± 2.7*y*/0.0%	Basketball (OP) college athletes with more than 10 years of basketball training and competition experience (E) vs. college students not participating in any team sports (*N*)	② Corsi block‐tapping task	0
Nakamoto and Mori (2008b)	*E*1 = 20/NC/0.0% *E*2 = 24/NC/0.0% *N *= 13/NC/0.0%	Basketball (OP) college athletes vs. baseball (OP) college athletes vs. sedentary college students (*N*)	① GO/NO GO (*E*1 = *E*2)	+&
Beavan et al. (2019)	*E*1 = 15/10.3 ± 0.6*y*/0.0% *E*2 = 17/11.2 ± 0.5*y*/0.0% E3 = 21/15.2 ± 0.3*y*/0.0% *E*4 = 21/16.7 ± 0.5*y*/0.0%	Soccer (OP) U12 athletes, 6.4 ± 1.7 years of training (*E*1) vs. soccer (OP) U13 athletes, 7.6 ± 1.7 years of training (*E*2) vs. soccer (OP) U17 athletes, 11.6 ± 2.5 years of training (*E*3) vs. soccer (OP) U19 athletes, 12.9 ± 2.2 years of training (*E*4)	① SST (*E*4 = *E*3 > *E*2 = *E*1)	&
Sakamoto et al. (2018)	*E*1 = 196/9.8 ± 1.1*y*/0.0% *E*2 = 187/9.6 ± 1.1*y*/0.0%	Players agreeing to join Japanese soccer (OP) League clubs (*E*1) vs. players refused to join Japanese soccer (OP) League clubs (*E*2)	① Stroop (*E*1 >* E*2) ③ Design fluency (*E*1 >* E*2)	& &
Lundgren et al. (2016)	*E*1 = 31/23.7 ± 5.0*y*/NC *E*2 = 19/23.7 ± 5.0*y*/NC	Ice Hockey (OP) elite athletes, all from the Swedish Ice Hockey A‐League (*E*1) vs. Ice Hockey (OP) sub‐elite athletes, all from the Swedish Ice Hockey B‐League (*E*2)	③ TMT‐B (*E*1 = *E*2)	&
Yu and Liu (2021)	*E*1 = 26/22.2 ± 1.5*y*/50.0% *E*2 = 26/21.7 ± 1.8*y*/53.8% *N *= 24/21.0 ± 2.0*y*/50.0%	Athletes with strategic skills (OP) such as soccer, basketball, etc., with an average of 6.1 years of training experience (*E*1) vs. athletes with blocking skills (OP) such as table tennis, tennis, etc., with an average of 5.6 years of training experience (*E*2) vs. non‐athletes (*N*)	① ANT (RT: *E*2 > *E*1; ACC: *E*1 >* E*2)	+&
Meng et al. (2019)	*E*1 = 35/22.7 ± 3.4*y*/34.3% *E*2 = 29/23.6 ± 2.8*y*/44.8% *N *= 27/22.8 ± 3.2*y*/63.0%	Badminton (OP) players with average 11.3 years of training experience (*E*1) vs. volleyball (OP) players with average 11.6 years of training experience (*E*2) vs. non‐athletes (*N*)	① SST (*E*2 > *E*1 = N) ① ANT (*E*1 = *E*2) ② CDT (*E*1 = *E*2) ③ TSWT (*E*1 = *E*2)	+0& 0& 0& +&
Wang et al. (2016)	*E *= 31/19–25*y*/35.5% *N *= 35/19–25*y*/40.0%	Table tennis (OP) level two or above athletes, more than 5 years of training experience, more than 3 times/week, and more than 2 h/session (*E*) vs. non‐athletes (*N*)	① ANT	+
Wang, Chang, Liang, Shih et al. (2013)	*E*1 = 14/20.6 ± 2.8*y*/0.0% *E*2 = 14/19.4 ± 0.7*y*/0.0% *N *= 14/21.2 ± 0.6*y*/0.0%	College tennis (OP) team member with 3–11 years of training experience (*E*1) vs. college swimming (CL) team member with 2.5–9 years of training experience (*E*2) vs. no sports experience (*N*)	① GO/NO GO (*E*1 > N; *E*2 = N)	+0&
Bashore et al. (2018)	*E *= 276/19.8 ± 1.5*y*/0.0% *N *= 32/19.6 ± 1.7*y*/0.0%	Soccer (OP) college athletes (*E*) vs. general students with no sports experience (*N*)	① Simon task	0
Bianco and Di Russo et al. (2017)	*E*1 = 13/29.4 ± 2.4*y*/38.5% *E*2 = 13/25.5 ± 1.5*y*/15.4% *N *= 13/28.5 ± 1.9*y*/23.1%	Fencing (OP) athletes, mean 11.2 years of experience in training (*E*1) vs. Boxing (OP) athletes, mean 11.7 years of experience in training (*E*2) vs. non‐athletes, 2.5 h/week physical activity (*N*)	① GO/NO GO (*E*1 = *E*2)	+&
Alves et al. (2013)	*E *= 87/16.3–24.9*y*/48.3% *N *= 67/16.5–23.3*y*/46.3%	Volleyball (OP) professional athletes (*E*) vs. non‐athletes (*N*)	① Stopping task ① Flanker ② Change detection ③ TSWT	+ 0 + +
Bianco and Berchicci et al. (2017)	*E *= 12/30.4 ± 6.2*y*/8.3% *N *= 12/28.8 ± 5.0*y*/8.3%	Fencing, table tennis, and volleyball (OP) athletes with an average of 13.4 years of training experience (*E*) vs. non‐athletes (*N*)	① GO/NO GO	+
Chang et al. (2017)	*E*1 = 20/21.2 ± 1.8*y*/30.0% *E*2 = 20/21.2 ± 1.2*y*/25.0% *N *= 20/21.6 ± 1.4*y*/35.0%	Marathon (CL) athletes (*E*1) vs. martial arts set (CL) athletes (*E*2) vs. non‐athletes (*N*)	① Stroop (*E*1 = *E*2) ③ WCST (*E*1 = *E*2) ③ Tower of London task (*E*1 = *E*2)	0& 0& 0&
Elferink‐Gemser et al. (2018)	*E*1 = 30/15.6 ± 3.6*y*/60.0% *E*2 = 30/15.9 ± 5.0*y*/60.0%	Table tennis (OP) elite athletes (*E*1) vs. table tennis (OP) sub‐elite athletes (*E*2)	① Stroop (*E*1 >* E*2) ③ Design fluency (*E*1 = *E*2) ③ TMT‐B (*E*1 = *E*2)	& & &
van de Water et al. (2017)	*E*1 = 15/25.0 ± 4.0*y*/0.0% *E*2 = 9/24.0 ± 4.0*y*/0.0%	Badminton (OP) elite athletes (*E*1) vs. badminton (OP) sub‐elite athletes (*E*2)	① SST (*E*1 = *E*2)	&
Wang et al. (2015)	*E *= 12/20.6 ± 2.8*y*/100.0% *N *= 13/19.1 ± 2.1*y*/100.0%	Badminton (OP) college athletes (*E*) vs. sedentary inactive control college students (*N*)	② Delayed match‐to‐sample test	+
Terpstra et al. (2019)	*E *= 61/55.0 ± 12.8*y*/0.0% *N *= 31/49.7 ± 12.1*y*/0.0%	Soccer (OP) retired athletes who played in the Canadian Football League (*E*) vs. controls with no exercise experience (*N*)	① GO/NO GO	0
Vestberg et al. (2020)	*E*1 = 23/24.4 ± 4.7*y*/59.4% *E*2 = 28/24.5 ± 4.6*y*/59.4%	Soccer (OP) national team players (*E*1) vs. soccer (OP) league players (*E*2)	③ Design fluency (*E*1 >* E*2)	&
Alarcón et al. (2017)	*E*1 = 12/25.2 ± 4.2*y*/0.0% *E*2 = 12/20.7 ± 0.8*y*/0.0% E3 = 12/22.7 ± 2.3*y*/0.0%	Basketball (OP) professional (*E*1) vs. basketball (OP) semi‐professional (*E*2) vs. basketball (OP) amateur (*E*3)	① Stroop (*E*1 = *E*2 = *E*3) ③ Design fluency (*E*1 >* E*2 = *E*3)	& &
Kruger et al. (2019)	*E*1 = 55/26.2 ± 3.8*y*/0.0% *E*2 = 24/22.2 ± 1.3*y*/0.0%	Rugby (OP) elite athletes (*E*1) vs. rugby (OP) sub‐elite athletes (*E*2)	② Delayed match‐to‐sample test (*E*1 = *E*2)	&
Rodrigues et al. (2019)	*E*1 = 44/24.6 ± 4.5*y*/0.0% *N *= 47/25.9 ± 4.2*y*/0.0%	Soccer (OP) professional athletes, both participating in the Brazilian Series A Championship (*E*) vs. non‐athletes (*N*)	① Stroop ② 2‐back ② Delayed memory test ③ Number‐letter‐test	0 + + +
Yu et al. (2017)	*E*1 = 18/21.1 ± 1.2*y*/44.4% *E*2 = 18/21.1 ± 2.0*y*/38.9% *N *= 18/21.8 ± 2.1*y*/50.0%	Badminton (OP) college athletes with an average of 11.3 years of training experience (*E*1) vs. track and field (CL) college athletes with an average of 7.9 years of training experience (*E*2) vs. college students who did not participate in any training (*N*)	① TSWT (*E*1 >* E*2 = N)	+0&
De Waelle et al. (2021)	*E*1 = 86/10.2 ± 1.0*y*/100.0% *E*2 = 25/10.3 ± 1.1*y*/100.0% *N *= 59/10.4 ± 1.1*y*/100.0%	Participants in soccer, basketball, volleyball, and field hockey (OP) with a training frequency of 2h/week or more (*E*1) vs. participants in cycling, swimming, and track and field (CL) with a training frequency of 2 h/week or more (*E*2) vs. students who do not participate in any exercise (*N*)	① Double trouble (*E*1 >* E*2 = N) ① Sustained attention to response (*E*1 >* E*2 = N) ② Monkey ladder (*E*1 >* E*2 = N) ② Spatial span (*E*1 > *E*2 = N) ② Token search (*E*1 > *E*2 = N) ③ Odd one out (*E*1 > *E*2 = N)	+0& +0& +0& +0& +0& +0&
Yongtawee et al. (2022)	*E*1 = 30/24.4 ± 2.4*y*/0.0% *E*2 = 30/24.9 ± 2.7*y*/0.0% *E*3 = 30/21.4 ± 2.1*y*/0.0% *N *= 30/24.8 ± 2.7*y*/0.0%	Boxing (OP) athletes (*E*1) vs. soccer (OP) athletes (*E*2) vs. shooting (CL) athletes (*E*3) vs. non‐athletes (*N*)	① Flanker (*E*1 = *E*2 = *E*3) ③ Design fluency (*E*1 = *E*2 > *E*3 = N) ③ TMT‐B (*E*1 = *E*2 = *E*3)	0& 0+& 0&
Shao et al. (2020)	*E*1 = 11/29.4 ± 0.7*y*/18.2% *E*2 = 9/17.9 ± 1.2*y*/33.3% *N *= 12/23.5 ± 0.3*y*/25.0%	Shooting (CL) elite athletes, all of whom participated in international competitions and excelled in national competitions, with an average of 18.13 years of training experience (*E*1) vs. shooting (CL) sub‐elite athletes, with an average of 2.84 years of training experience (*E*2) vs. control group with no training experience (*N*)	① Flanker (*E*1 = *E*2)	+&
Stacey et al. (2021)	*E *= 11/30.0 ± 8.0*y*/0.0% *N *= 11/30.0 ± 10.0*y*/0.0%	Brazilian Jiu‐Jitsu (OP) athletes (*E*) vs. non‐athletes (*N*)	② RBD ③ TMT‐B	0 0
Vaughan and Laborde (2021)	*E*1 = 81/18.9 ± 1.0*y*/54.9% *E*2 = 87/18.9 ± 1.0*y*/54.9% *E*3 = 92/18.9 ± 1.0*y*/54.9% *N *= 99/18.9 ± 1.0*y*/54.9%	Basketball (OP) super elite players (*E*1) vs. Basketball (OP) elite players (*E*2) vs. basketball (OP) amateur players (*E*3) vs. basketball (OP) novice players (*N*)	② Corsi blocks task (*E*1 > *E*2 > *E*3 > N) ② SWM (*E*1 > *E*2 > *E*3 > N)	+& +&
Seo et al. (2012)	*E *= 20/28.9 ± 7.3*y*/100.0% *N *= 23/26.3 ± 4.6*y*/100.0%	Archery (CL) outstanding athlete with more than 10 years of competition experience (*E*) vs. non‐athletes (*N*)	② JLO	0
Verburgh et al. (2014)	*E*1 = 69/10.6 ± 1.4*y*/0.0% *E*2 = 48/10.5 ± 1.3*y*/0.0% *N *= 51/10.4 ± 1.2*y*/0.0%	Soccer (OP) elite athletes, all from youth academies of Dutch professional soccer clubs (*E*1) vs. soccer (OP) sub‐elite athletes, all from amateur soccer clubs (*E*2) vs. non‐athletes who did not take part in any exercise (*N*)	① SST(*E*1 > *E*2 = N) ① ANT(*E*1 = *E*2 = N) ② Digit Span Backwards (*E*1 = *E*2>N) ② VSTM (*E*1 = *E*2>N)	+0& 0& +& +&
Liao et al. (2017)	*E *= 42/22.7 ± 3.6*y*/33.3% *N *= 15/26.1 ± 2.63*y*/53.3%	Badminton (OP) athletes, both elite A players (*E*) vs. non‐athletes (*N*)	① SST	0
Cona et al. (2015)	*E*1 = 15/42.8 ± 9.6*y*/0.0% *E*2 = 15/42.1 ± 7.7*y*/0.0%	Marathon (CL) elite athletes (*E*1) vs. marathon (CL) sub‐elite athletes (*E*2)	① GO/NO GO (*E*1 = *E*2)	&
Wylie et al. (2018)	*E *= 280/19.9 ± 1.6*y*/0.0% *N *= 35/20.3 ± 2.6*y*/0.0%	Soccer (OP) college athletes (*E*) vs. controls with no soccer experience (*N*)	① Flanker	0
Gökçe et al. (2021)	*E*1 = 18/20.4 ± 1.9*y*/50.0% *E*2 = 18/21.0 ± 2.0*y*/50.0% *N *= 18/22.3 ± 1.9*y*/38.9%	Fencing (OP) athletes (*E*1) vs. swimming (CL) athletes (*E*2) vs. controls without regular exercise (*N*)	② Corsi block‐tapping task (*E*1 >* E*2 = *N*)	+0&

*Note*: Abbreviations and & indicate a comparison between exposed groups that requires further explanation; + indicates in favor of exposed group; 0 indicates insignificant difference between exposed and non‐exposed groups; ① = inhibitory control; ② = working memory; ③ = cognitive flexibility; —indicates in favor of non‐exposed group.

Abbreviations: ANT, Attention Network Task; CDT, Change Detection Task; CL, closed skills; CTT, Color Connections Test; *E*, exposed group; *F*%, percentage of females; JLO, Judgment of Line Orientation task; *N*, non‐exposed group; NC, not clear; OP, open skills; SST, Signal Stop Task; SWM, Spatial Working Memory Task; TMT‐B, Trail Making Test‐B; TST, Task Switching Task; VMS, Visual Memory Spread Task; VSTM, Visual Short‐Term Memory Task; WCST, Wisconsin Card Sorting Test; *y*, years.

#### 3.2.4 Test Instruments and Main Findings

A total of 32 (78.05%) studies reported inhibitory control; 14 (34.15%) studies reported working memory; and 16 (39.02%) studies reported cognitive flexibility.

In the tools for inhibitory control tests, the GO/NO GO, Stroop, Attention Network Task, and Flanker tasks are common test tasks. Among them, eight studies (Bianco and Berchicci et al. 2017; Bianco and Di Russo et al. 2017; Chan et al. 2011; Cona et al. 2015; Nakamoto and Mori 2008a, 2008b; Terpstra et al. 2019; Wang, Chang, Liang, Shih et al. 2013) used the GO/NO GO task for testing; seven studies (Alarcón et al. 2017; Chang et al. 2017; Elferink‐Gemser et al. 2018; Rodrigues et al. 2019; Sakamoto et al. 2018; Vestberg et al. 2012; Wu et al. 2022) used the Stroop task for testing; six studies (Beavan et al. 2019; Huijgen et al. 2015; Liao et al. 2017; Meng et al. 2019; van de Water et al. 2017; Verburgh et al. 2014; Wang, Chang, Liang et al. 2013) used the Attention Network Task for testing; 4 studies (Alves et al. 2013; Shao et al. 2020; Wylie et al. 2018; Yongtawee et al. 2022) used the Flanker task for testing. Additionally, in the 45 cross‐comparisons of inhibitory control tests between athletes and a general control group, 22 studies demonstrated that athletes have a more active inhibitory control advantage. In the 25 cross‐comparisons of inhibitory control tests among athletes of different levels, 13 studies showed that athletes of a higher level exhibit positive inhibitory control behaviors.

In the tools for working memory tests, the Corsi Blocks Task, Delayed Memory Test, and N‐back are the most common assessment tools. Among them, three studies (Furley and Memmert 2010; Gökçe et al. 2021; Vaughan and Laborde 2021) used the Corsi Blocks Task for assessment; three studies (Kruger et al. 2019; Rodrigues et al. 2019; Wang et al. 2015) used the Delayed Memory Test for assessment; and two studies (Rodrigues et al. 2019; Wu et al. 2022) used the N‐back task for assessment. In addition, in the 27 cross‐comparisons of working memory tests between athletes and general control groups, 18 studies showed that athletes had higher working memory performance. In the 12 cross‐comparisons of working memory tests among athletes of different levels, 7 studies indicated that athletes of higher levels had more positive working memory performance.

In the tools for assessing cognitive flexibility, the Trail Making Test‐B and Design Fluency Task are the most commonly used assessment tools. Among them, six studies (Elferink‐Gemser et al. 2018; Huijgen et al. 2015; Lundgren et al. 2016; Stacey et al. 2021; Vestberg et al. 2012; Yongtawee et al. 2022) used the Trail Making Test‐B for assessment; six studies (Alarcón et al. 2017; Elfrink‐Gemser et al. 2018; Sakamoto et al. 2018; Vestberg et al. 2012, 2020; Yongtawee et al. 2022) used the Design Fluency Task for assessment. In addition, in the 20 cross‐comparisons of cognitive flexibility tests between athletes and general control groups, 10 studies showed that athletes had more active cognitive flexibility; in the 17 cross‐comparisons among athletes of different levels, 9 studies indicated that the higher the level of the athlete, the higher their cognitive flexibility. See Table 1 for more descriptive information on participants, exposure and control measures, measurement of outcome variables, and results.

### 3.3 Quality Assessment Results

All 41 articles reported the source of the information, the age stage of the participants, and the completeness of the data collection. Twenty‐three (56.10%) of the included studies described assessments to assure the quality of the study, such as controlling for demographic information such as age, gender, and educational level of the participants, as well as physical morphologic information such as height, weight, and BMI. Twenty (48.78%) articles described measures to control confounders, which were implemented primarily during the screening phase of participants. Fewer studies (21.95%) reported in detail the inclusion and exclusion criteria for the exposed and control groups. Given the completeness of the data collected by the larger number of studies, there were few studies explaining the reasons for exclusion of participants (19.51%) and explaining the handling of missing data (17.07%). In addition, it is not clear whether the researcher's subjective factors masked the specifics of the participants (Table 2).

**TABLE 2:** Results of quality assessment of the included studies.

Included studies	⑴	⑵	⑶	⑷	⑸	⑹	⑺	⑻	⑼
Wu et al. (2022)	①	②	①	③	②	①	①	②	①
Yu et al. (2019)	①	①	①	③	①	③	①	③	①
Huijgen et al. (2015)	①	②	①	③	②	②	②	③	①
Chan et al. (2011)	①	②	①	③	①	②	②	③	①
Nakamoto and Mori (2008a)	①	②	①	③	②	③	②	③	①
Vestberg et al. (2012)	①	②	①	③	①	③	②	③	①
Wang, Chang, Liang et al. (2013)	①	①	①	③	①	③	①	①	①
Furley and Memmert (2010)	①	②	①	③	②	③	②	③	①
Nakamoto and Mori (2008b)	①	②	①	③	②	②	②	③	①
Beavan et al. (2019)	①	②	①	③	②	②	②	③	①
Sakamoto et al. (2018)	①	②	①	③	①	②	①	③	①
Lundgren et al. (2016)	①	②	①	③	①	①	①	①	①
Yu and Liu (2021)	①	②	①	③	③	③	②	③	①
Meng et al. (2019)	①	①	①	③	①	①	①	①	①
Wang et al. (2016)	①	②	①	③	①	③	①	③	①
Wang, Chang, Liang, Shih et al. (2013)	①	②	①	③	①	①	①	①	①
Bashore et al. (2018)	①	②	①	③	①	①	①	①	①
Bianco and Di Russo et al. (2017)	①	①	①	③	①	③	①	③	①
Alves et al. (2013)	①	①	①	③	②	①	②	③	①
Bianco and Berchicci et al. (2017)	①	②	①	③	②	③	②	③	①
Chang et al. (2017)	①	②	①	③	①	①	①	①	①
Elferink‐Gemser et al. (2018)	①	②	①	③	①	③	①	③	①
van de Water et al. (2017)	①	②	①	③	①	③	②	③	①
Wang et al. (2015)	①	②	①	③	①	③	①	③	①
Terpstra et al. (2019)	①	①	①	③	①	③	①	③	①
Vestberg et al. (2020)	①	②	①	③	②	③	②	③	①
Alarcón et al. (2017)	①	②	①	③	②	②	②	②	①
Kruger et al. (2019)	①	②	①	③	②	②	①	③	①
Rodrigues et al. (2019)	①	②	①	③	①	③	①	③	①
Yu et al. (2017)	①	②	①	③	①	③	①	①	①
De Waelle et al. (2021)	①	②	①	③	②	②	②	②	①
Yongtawee et al. (2022)	①	②	①	③	①	③	①	②	①
Shao et al. (2020)	①	②	①	③	②	③	②	②	①
Stacey et al. (2021)	①	②	①	③	①	②	①	②	①
Vaughan and Laborde (2021)	①	①	①	③	②	③	②	②	①
Seo et al. (2012)	①	②	①	③	②	②	②	②	①
Verburgh et al. (2014)	①	②	①	③	①	①	①	②	①
Liao et al. (2017)	①	②	①	③	①	②	③	②	①
Cona et al. (2015)	①	①	①	③	①	②	②	②	①
Wylie et al. (2018)	①	②	①	③	②	③	②	②	①
Gökçe et al. (2021)	①	①	①	③	②	③	②	②	①

*Note*: (1) Are the sources of information identified? (2) Are inclusion and exclusion criteria for exposed and non‐exposed groups listed or references to previous publications? (3) Is the time period for identifying patients given? (4) Did the evaluator's subjective factors overshadow other aspects of the study population? (5) Describes any assessment for quality assurance; (6) explains the rationale for excluding any patients from the analysis; (7) describes how confounders were evaluated and/or measures to control for them; (8) explains how missing data were handled in the analysis, if possible; (9) summarizes the response rate of the patients and the completeness of the data collection; ① = yes; ② = no; ③ = not clear.

### 3.4 Comparative Analysis of Executive Functions Across Different Groups

Combined effects tests (Table 3) based on a random‐effects model (*I* ^2^ > 85%, *p* < 0.001) showed that athletes showed more positive inhibitory control (*Z *= 5.18, *SMD *= −0.631, 95%*CI *= −0.869 to −0.392, *p *= 0.000) and working memory (*Z *= 3.42, *SMD *= −0.382, 95%*CI *= −0.601 to −0.163, *p *= 0.001). However, there is no significant difference between the two groups in terms of cognitive flexibility (*Z *= 1.95, *SMD *= −0.377, 95%*CI *= −0.755 to 0.002, *p *= 0.051). As a result, the athletes had superior executive functions compared to the general group, especially in inhibitory control and working memory. Thus, research hypothesis H1 was confirmed.

**TABLE 3:** Quantitative comparative results of executive functions between athletes and the general group.

Outcome variables	Heterogeneity test		Combined effects test			
	*I* ^2^ (%)	*p*	*Z*	*p*	*SMD*	95%*CI*
Inhibitory control	89.0	0.000	5.18	0.000	−0.631	(−0.869, −0.392)
Working memory	87.7	0.000	3.42	0.001	−0.382	(−0.601, −0.163)
Cognitive flexibility	89.7	0.000	1.95	0.051	−0.377	(−0.755, 0.002)

*Notes*: Some of the included studies were multi‐arm studies, that is, involving athletes engaged in different types of skill training, which were split into independent studies according to the Cochrane Handbook (Zhang et al. 2009). Some of the included studies used multiple indictors to reflect inhibitory control, working memory, or cognitive flexibility in participants, and the studies were included in the meta‐analysis for combined effects tests. The same caveats apply to the figures for the subsequent analyses.

Abbreviations: CI, confidence intervals; SMD, standardized mean difference.

There was no large heterogeneity between studies comparing working memory in sub‐elite and amateur athletes (*I* ^2^ = 45.2%, *p *= 0.177), so the analysis was conducted using a fixed‐effects model, whereas there was large heterogeneity for the remaining indicators (*I* ^2^ > 60%, *p* < 0.05), so the analysis was conducted using a random‐effects model. The results of the combined effects test (Table 4) showed that elite compared to sub‐elite athletes performed more positively on the cognitive flexibility task (*Z *= 2.89, *SMD *= −0.389, 95%*CI *= −0.652 to −0.125, *p *= 0.004), but there was no significant difference in performance on the inhibitory control and working memory tasks (*p *> 0.05); sub‐elite compared to sub‐elite athletes performed more positively on the cognitive flexibility task (*Z *= 2.01, *SMD *= −0.740, 95%*CI *= −1.462 to −0.017, *p *= 0.005), but there was no significant difference in performance on the inhibitory control and working memory tasks (*p *> 0.05). Thus, as athletes move up the athletic hierarchy and accumulate athletic experience, their executive functions become more superior, especially in terms of cognitive flexibility, and research hypothesis H2 is supported.

**TABLE 4:** Quantitative comparative results of executive function in elite, sub‐elite, and amateur athletes.

Comparison between groups	Outcome variables	Heterogeneity test		Combined effects test			
		*I* ^2^ (%)	*p*	*Z*	*p*	*SMD*	95%*CI*
Elites and sub‐elites	Inhibitory control	69.8	0.003	0.85	0.393	−0.152	(−0.501, 0.197)
	Working memory	83.6	0.014	0.12	0.908	0.047	(−0.746, 0.839)
	Cognitive flexibility	64.0	0.005	2.89	0.004	−0.389	(−0.652, −0.125)
Sub‐elites and amateurs	Inhibitory control	68.2	0.004	1.66	0.094	−0.342	(−0.744, 0.061)
	Working memory	45.2	0.177	1.95	0.051	−0.294	(−0.589, 0.001)
	Cognitive flexibility	81.1	0.005	2.01	0.045	−0.740	(−1.462, −0.017)

Abbreviations: CI, confidence intervals; SMD, standardized mean difference.

### 3.5 Differences in Executive Functions Between Open‐ and Closed‐Skill Athletes

There was no significant inter‐study heterogeneity in the comparison of working memory between the closed‐skilled athletes and the general group (*I* ^2^ = 24.6%, *p *= 0.258), so the analysis was conducted using a fixed‐effects model, whereas there was significant heterogeneity in the rest of the indicators (*I* ^2^ > 55%, *p* < 0.05), so the analysis was conducted using a random‐effects model. The results of the combined effects test (Table 5) showed that open‐skilled athletes performed more positively compared to the general group in inhibitory control (*Z *= 4.55, *SMD *= −0.660, 95%*CI *= −0.950 to −0.369, *p *= 0.000), working memory (*Z *= 3.62, *SMD *= −0.455, 95%*CI *= −0.702 to −0.208, *p *= 0.000), and cognitive flexibility (*Z *= 2.07, *SMD *= −0.595, 95%*CI *= −1.160 to −0.031, *p *= 0.039); closed‐skill athletes performed more positively in inhibitory control compared to the general group (*Z *= 2.88, *SMD *= −0.524, 95%*CI *= −0.881 to −0.167, *p *= 0.004), but there was no significant difference in working memory and cognitive flexibility (*p *> 0.05). Therefore, there are skill‐type differences in executive functions of athletes, meaning that open‐skilled athletes have superior executive functions, and research hypothesis H3 is supported.

**TABLE 5:** Quantitative comparative results of executive function in different types of skilled athletes and the general group.

Skill types	Outcome variables	Heterogeneity test		Combined effects test			
		*I* ^2^ (%)	*p*	*Z*	*p*	*SMD*	95%*CI*
Open skills	Inhibitory control	90.9	0.000	4.55	0.000	−0.660	(−0.950, −0.369)
	Working memory	89.2	0.000	3.62	0.000	−0.455	(−0.702, −0.208)
	Cognitive flexibility	92.8	0.000	2.07	0.039	−0.595	(−1.160, −0.031)
Closed skills	Inhibitory control	72.8	0.000	2.88	0.004	−0.524	(−0.881, −0.167)
	Working memory	24.6	0.258	0.07	0.944	−0.010	(−0.279, 0.259)
	Cognitive flexibility	55.6	0.028	0.38	0.701	−0.060	(−0.366, 0.246)

Abbreviations: CI, confidence intervals; SMD, standardized mean difference.

### 3.6 Publication Bias Test

The reliability of the results of the meta‐analysis depends on the presence of bias in the included studies. This study used Egger's linear regression for publication bias testing. Egger's linear regression is a quantitative test for the presence of publication bias to compensate for the shortcomings of funnel plots when they are subjectively unable to determine the situation (Shi, Li et al. 2022). In this study, the effect sizes of the comparisons of inhibitory control, working memory, and cognitive flexibility between athletes and controls, respectively, were used as the dependent variables, and the precision of the effect estimates was used as the independent variable to construct linear regression equations, and the intercept of the regression equations was the offset, which was closer to 0, indicating that there was a lower likelihood of publication bias, and that there was no publication bias if the *p *> 0.05 and the 95%*CI* contained 0 (Shi et al. 2022). The results in Table 6 show that *p *< 0.05 and 95%*CI* does not contain 0 in inhibitory control, thus suggesting that there may be publication bias in the included studies; however, *p *> 0.05 and 95%*CI* contains 0 in working memory and cognitive flexibility, thus indicating that there is no publication bias in the included studies, and the results of the meta‐analysis are stable and reliable.

**TABLE 6:** Publication bias test for Egger's linear regression.

Outcome variables	*β*	*SE*	*t*	*p*	95%*CI*
Inhibitory control	−5.195	1.251	−4.15	0.000	(−7.713, −2.677)
Working memory	−0.517	2.081	−0.25	0.806	(−4.803, 3.770)
Cognitive flexibility	2.309	3.600	0.64	0.530	(−5.287, 9.905)

Abbreviation: CI, confidence intervals.

### 3.7 Sources of Heterogeneity

**FIGURE 2:**
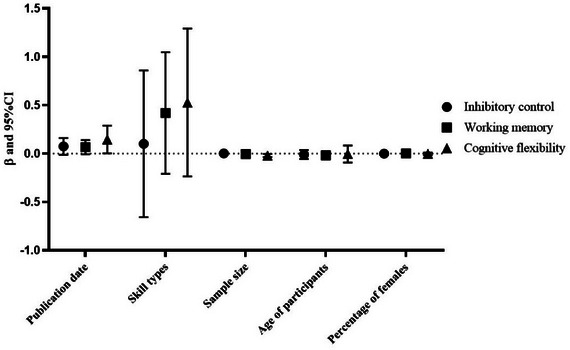
Results of one‐way meta‐analysis. CI, confidence intervals.

The results of the heterogeneity test showed a high level of heterogeneity among the included studies, so a one‐way meta‐regression analysis of the characteristics of the studies that may have caused the heterogeneity was needed to explore the sources of inter‐study heterogeneity (Zhang et al. 2009). In this study, the effect sizes of inhibitory control, working memory, and cognitive flexibility comparisons between athletes and controls, respectively, were assigned and coded for publication date, skill type, sample content, age of participants, and proportion of females, which were set as independent variables for one‐way meta‐regression analyses. The results (Figure 2) showed that publication date (*β *= 0.145) and sample size (*β *= −0.002) were sources of heterogeneity among the included studies of cognitive flexibility (*p *< 0.05).

### 3.8 Sensitivity Analysis

This study was conducted with the help of “metaninf” command for the sensitivity analysis of one‐by‐one exclusion method. After excluding a study one by one, the SMD for inhibitory control was −0.655 to −0.475, 95%*CI *=  (−0.893 to −0.641, −0.414 to −0.309), the SMD for working memory was −0.409 to −0.290, 95%*CI *=  (−0.630 to −0.444, −0.180 to −0.131), and the SMD for cognitive flexibility was −0.438 to −0.229, 95%*CI *=  (−0.819 to −0.458, −0.010 to 0.054). Thus, for inhibitory control and working memory, none of the results of the combined effects tests after excluding a study one by one were substantially changed; however, for cognitive flexibility, there may be a literature sensitivity issue. In addition, this study used the cut‐and‐patch method proposed by Rothstein et al. (2005) to identify and correct funnel plot asymmetry caused by publication bias, and the results showed that no new studies were added after the cut‐and‐patch, and the results did not change, so the results were robust and reliable (Buscemi et al. 2006).

## 4 Discussion

### 4.1 Athletes Have Superior Executive Functions

The results of this study showed that the athletes had superior executive function compared to the general group, a result similar to the studies by Logan et al. (2023) and Scharfen and Memmert (2019). Motor skills are the basis and prerequisite for participation in physical exercise (Stodden et al. 2008), and athletes have higher sports skill performance compared to the general group and, therefore, are able to achieve more cognitive benefits from physical exercise. Relevant systematic reviews and meta‐analyses (Feng et al. 2023; Gu et al. 2019; Heilmann et al. 2022) have also confirmed that long‐term exercise based on motor skill learning has a significant promoting effect on executive functions, which provides some support for the association between excellent skill performance and executive functions.

From a neurophysiological point of view, long‐term training induces plasticity changes in the physiological structure and functions of the central nervous system, synapses, and cells of the brain (Sagi et al. 2012), improves the morphological structure and functional activity of the brain (Vints et al. 2022; Zatorre et al. 2012), and, therefore, exhibits superior executive functions. In addition, the learning of sport skills in stationary or changing situations contributes to the accumulation of specific cognitive experiences, whereas sport skills learning and executive function tasks have brain regions activated in prefrontal cortex areas, and enhancing sport skills has facilitative benefits for motor execution, decision‐making, and attentional information processing (Cao et al. 2017).

From the perspective of sports experience, athletes benefiting from the accumulated action experience from long‐term sport skills learning tend to decrease the activation of brain regions in action anticipation and action execution tasks and increase the activity of brain regions related to cognitive comprehension such as frontal, parietal, and occipital lobes, which facilitates the functional connectivity of these brain regions, which, in turn, improves visual attention, perceptual anticipation, and executive functions (Huang et al. 2015; Jacini et al. 2009; Wu et al. 2022). Xu (2019) explored the behavioral and EEG characteristics of table tennis players and college students in a Go/NoGo task and found that the athletes’ response time and accuracy were significantly higher than those of college students in the Go stimulus. In addition, the study (Xu 2019) found that athletes exhibited significant “NoGo effects” in the N2 and P3 components. Thus, the long‐term learning and training of sport skills has led to an increase in the experience accumulated by athletes, as well as an improvement in central task processing patterns and executive functions.

### 4.2 Sports Experience Is Strongly Associated With Executive Function

The results of this study show that elite athletes have significantly higher cognitive flexibility than sub‐elite athletes, and sub‐elite athletes have significantly higher cognitive flexibility than amateur athletes. This implies that as the level of sports proficiency increases and sports experience accumulates, athletes’ cognitive flexibility is enhanced, further validating the close link between sports experience and executive function. The results of this study showed that as sport level increased, executive function of athletes also increased, especially in cognitive flexibility, further validating the close link between sports experience and executive function. Cognitive flexibility is a more complex skill in executive functions, relative to inhibitory control and working memory, and all conscious attentional control and shifting relies on the development of inhibitory control and working memory and their coordination with each other (Diamond 2013). Shi and Tang et al. (2022) confirmed that cognitive flexibility requires a longer intervention time to achieve more positive enhancement effects compared to inhibitory control and working memory. Thus, the development of cognitive flexibility may require more sports experience.

Sports experience is accumulated due to the repetitive practice of multi‐limbed movements and cognitive challenges stimulated by sports scenarios. Motor skills are temporary neural connections established in the relevant centers of the cerebral cortex with the participation of multiple sensory organs (Dayan and Cohen 2011), and in particular, the repetitive practice of multi‐limbed movements helps to activate the relevant neural pathways and promotes the development of executive functions (Diamond 2015). Therefore, accumulating experience in complex movements can promote the development of executive functions. In addition, the rich variation of stimuli in the external environment of open skills constantly exposes individuals to new problems and challenges. Individuals need to coordinate and combine their original actions, integrate their existing declarative knowledge experiences to make more rational coordinated responses, or create more novel actions, so that the relevant neural circuits in the cerebral cortex continue to establish connections and, thus, continue to improve their cognitive processes (Kolb and Gibb 2011; Pellis and Pellis 2007).

In addition, some researchers have verified the association between sports experience and executive functions using training frequency. For example, Moratal et al. (2020) found that 8–12‐year‐olds who regularly participated in soccer training had more pronounced strengths in cognitive tasks such as working memory, attention, and information processing speed. Ishihara et al. (2017a, 2017b) assessed the relationship between frequency of tennis training and executive functions in children and adolescents, and after controlling for age, gender, BMI, and tennis experience, more frequent tennis training was associated with higher processing speed and inhibitory control in boys and with better working memory in boys and girls. A review by Paiano et al. (2019) found that adolescent athletes in professional soccer schools had better executive functions than those in amateur schools. In summary, more frequent training accumulates more sports experience and, thus, yields greater benefits for executive function.

### 4.3 Differences in Skill Types Exist in Executive Functions of Athletes

The results of this study showed that there were skill‐type differences in executive function, that is, athletes who participated in open skills training had more positive executive functions, especially in working memory and cognitive flexibility. Sport skill acquisition includes cognitive, associative, and automatic phases. Individuals, in the early stages of skill learning, inhibit unreasonable visual motor plans and assess new audiovisual and kinesthetic information through the working memory system, activating specific frontal lobe areas (Gentili et al. 2013), but as motor skills reach an automated level, the activation of the frontal lobe areas responsible for inhibition and refreshing decreases (Sakai et al. 1998). However, athletes engaged in open skills training are faced with dynamic and variable stimulus information, and the completion of their technical movements is not automated, requiring selective attention from the audiovisual perceptual system to achieve technical selection and decision‐making (Tomporowski and Pesce 2019). Thus, the complexity of cognitive stimuli may account for the differences in the types of skills that exist in executive functions in athletes.

In addition, several studies (Koutsandreou et al. 2016; Shi et al. 2022) have shown that both open‐ and closed‐skill training significantly contribute to executive functions. However, given the different effects of different types of skills on brain organization and neural activation, there are differences in the outward manifestation of executive control. In particular, closed skills, such as jogging, cycling, and aerobics, increase brain tissue capillary density and activate sensory‐motor networks that regulate response inhibition (Voelcker‐Rehage et al. 2011), whereas open skills, such as basketball and table tennis, promote individuals’ perceptual‐motor coordination, increase the number of Purkinje neurons and synapses, and activate visuospatial networks associated with attentional control and working memory (Mavilidi et al. 2015). In addition, open skills involve richer environmental and interpersonal interaction stimuli that better induce neural remodeling and increase neural efficiency (Mavilidi et al. 2015; Moreau et al. 2015). Therefore, athletes with open skills have superior executive function behavioral performance.

### 4.4 Limitations of This Study

First, the search process is limited by language, which may have led to publication bias. However, the sensitivity analyses of the one‐by‐one culling method and the cut‐and‐patch method showed robust and reliable results, and the findings were similar to those of similar published studies (Logan et al. 2023; Scharfen and Memmert 2019). Second, the included studies may have methodological quality issues that may interfere to some extent with the accuracy of the findings. Third, previous studies have indirectly reflected sports experience in terms of level of sports proficiency, years of training, whereas sports experience is not fully and positively correlated with level of sports proficiency, years of training, and there are no studies that have used relevant instruments to directly measure sport skills performance, so there is also a lack of direct evidence. Finally, due to the relative scarcity of studies on amateur players and control groups included in this research, this study has not yet conducted comparisons between amateur players and control groups. We also look forward to future research to further accumulate primary studies to confirm this. In addition, most current studies use CSS to compare the executive functions of elite, sub‐elite, amateur, and general control groups, in order to examine the potential role of executive functions in sports experience. However, cross‐sectional studies cannot establish a true causal relationship, so subsequent research using longitudinal studies is needed for further exploration.

## 5 Conclusion

This study, through a combined effects test of 41 studies exploring executive functions in athletes, found that athletes have higher executive functions, particularly in inhibitory control and working memory, compared to the general population with no sports experience. In addition, elite compared to sub‐elite athletes performed more positively on a cognitive flexibility task; sub‐elite compared to amateur athletes performed more positively on a cognitive flexibility task, that is, the cognitive flexibility was superior as they moved up the level of sports proficiency and gained sports experience. Subgroup analyses found that open‐skill athletes outperformed the general population without sports experience in inhibitory control, working memory, and cognitive flexibility, whereas closed‐skill athletes performed more positively only in inhibitory control.

The above research findings provide psychological guidance for the selection and development of athletes. First, coaches can use executive function test systems to select athletes with higher executive function performance for focused training. This is because such individuals often possess higher brain functions, which enable them to demonstrate complex body control and flexible tactical decision‐making in sports performance. Second, researchers can intervene at the cognitive and psychological levels to address certain executive function behavioral deficits in athletes, in order to enhance their executive functions and thereby promote improvements in their sports performance.

## Author Contributions

S.R. and P.S. wrote the manuscript. K.Z. and W.W. proofread the manuscript. X.F., K.Z., W.W., and P.S. searched for relevant original studies and performed screening, coding, and other tasks. P.S. provides ideas for topic selection and guidance for revision. All authors participated in the intellectual content of the manuscript.

## Ethics Statement

The authors have nothing to report.

## Conflicts of Interest

The authors declare no conflicts of interest.

### Peer Review

The peer review history for this article is available at https://publons.com/publon/10.1002/brb3.70212

## Data Availability

All data generated or analyzed during this study are included in this published article.
